# The X-Ray Treatment of Inoperable Carcinoma of the Breast; Report of a Case

**Published:** 1904-03

**Authors:** James N. Brawner

**Affiliations:** Atlanta, Ga.


### THE X-RAY TREATMENT OF INOPERABLE CARCINOMA OF THE BREAST; REPORT OF A CASE.*

By JAMES N BRAWNER, M.D.,

ATLANTA, GA.

*Mr. President and Gentlemen:*

The case which I wish to show you to-night is of interest because it shows the benefit that can be derived from the use of the X-ray in these cases, and at the same time it shows that a permanent cure need not be expected.

This lady is about forty-five years of age, has several children, has always been fairly healthy, and gives no history of cancer in any of her relatives. About fourteen years ago she received a severe bruise of the right breast which, she states, caused an abscess to form. She had a baby seven months old at this time. Two years ago a nodule began to form in the site of the old abscess, which grew larger and larger until it consumed a considerable part of the breast. The axillary glands also became involved. Last February, or about eleven months ago, she consented to have an operation performed, which was done by Dr. Monroe Smith. He removed all of the breast, much of the pectoralis muscle, and the axillary glands. Skin-grafting was resorted to in order to cover the wound. About six months after the operation, recurrence occurred in the second and third ribs about two inches from the sternum. Recurrence also occurred in the scar, the skin breaking down in several places. At this time the apex of the right lung seemed to be consolidated. As soon as this condition was noticed the X-ray treatment was advised and I began the treatment about

* Presented at the meeting of February 4 of the Atlanta Society of Medicine.

October 1, 1903, and have continued it from that time, a period of about four months.

At first I used lead foil to protect other portions of the body, but since then have used nothing, allowing the rays to penetrate all the tissues near the carcinomatous growth. Since commencing the X-ray treatment, the enlargement of the second and third ribs has diminished considerably, the ulcers recurring in the old scar have also almost entirely healed. The ulcers at times appear to be perfectly well, then they show a tendency to break down and then heal up again under the influence of the rays. At present the consolidation of the lung tissue is worse than it was three months ago, showing that the disease is advancing in this region. During the last month, the pericardium seems to be involved, as there are a few pericardial friction sounds to be heard over the left fourth intercostal space. The sounds are heard only on pressure of the heart or during inspiration. It is impossible to determine whether the mediastinum is involved or not. The patient has gained six pounds in weight since beginning the X-ray treatment.

It is plain to any one, considering this case, that the X-rays have been of great value in prolonging the patient's life and adding to her comfort.

---

### PERUNA.

*Editor Medical World:* I noticed the question about Peruna in your last issue. In a drug-store, where I fixed up a laboratory, an imitation of Peruna was manufactured and sold. This imitation was composed of:

| | | |
|---|---|---|
| Comp. fl. ext. of gentian | | ʒ 90 |
| Fl. ex. of taraxacum | cong. j | ℥ 52 |
| Acid phos. dil. | | ʒ 75 |
| Tr. cardamon comp. | | ʒ 90 |
| Syrup simplex | cong. ij | ℥ 104 |
| Glycerin | cong. vij | ʒ iv |
| Sherry wine | cong. ix | ℥ 108 |
| Trit. { Aloin | | ʒ iv |
| Trit. { Cascarin | | ℥ ij gr. 120 |

This imitation appeared to me to be a very clever one; if anything, it seems to be an improvement on Peruna, so far at least as sherry wine is preferable to whisky. I. LADOFF.

Schenectady, N. Y.

